# Interobserver Variability in Manual Versus Semi-Automatic CT Assessments of Small Lung Nodule Diameter and Volume

**DOI:** 10.3390/tomography10120148

**Published:** 2024-12-19

**Authors:** Frida Zacharias, Tony Martin Svahn

**Affiliations:** 1 Department of Imaging and Functional Medicine, Division Diagnostics, Hudiksvall Hospital, Region Gävleborg, SE 824 81 Hudiksvall, Sweden; frida.zacharias@regiongavleborg.se; 2 Centre for Research and Development, Uppsala University, Region Gävleborg, SE 801 88 Gävle, Sweden; 3 Department of Imaging and Functional Medicine, Division Diagnostics, Gävle Hospital, Region Gävleborg, SE 801 88 Gävle, Sweden

**Keywords:** interobserver variability, semi-automatic measurement, computed tomography (CT), lung nodule size

## Abstract

Background: This study aimed to assess the interobserver variability of semi-automatic diameter and volumetric measurements versus manual diameter measurements for small lung nodules identified on computed tomography scans. Methods: The radiological patient database was searched for CT thorax examinations with at least one noncalcified solid nodule (∼3–10 mm). Three radiologists with four to six years of experience evaluated each nodule in accordance with the Fleischner Society guidelines using standard diameter measurements, semi-automatic lesion diameter measurements, and volumetric assessments. Spearman’s correlation coefficient measured intermeasurement agreement. We used descriptive Bland–Altman plots to visualize agreement in the measured data. Potential discrepancies were analyzed. Results: We studied a total of twenty-six nodules. Spearman’s test showed that there was a much stronger relationship (*p* < 0.05) between reviewers for the semi-automatic diameter and volume measurements (avg. r = 0.97 ± 0.017 and 0.99 ± 0.005, respectively) than for the manual method (avg. r = 0.91 ± 0.017). In the Bland–Altman test, the semi-automatic diameter measure outperformed the manual method for all comparisons, while the volumetric method had better results in two out of three comparisons. The incidence of reviewers modifying the software’s automatic outline varied between 62% and 92%. Conclusions: Semi-automatic techniques significantly reduced interobserver variability for small solid nodules, which has important implications for diagnostic assessments and screening. Both the semi-automatic diameter and semi-automatic volume measurements showed improvements over the manual measurement approach. Training could further diminish observer variability, given the considerable diversity in the number of adjustments among reviewers.

## 1. Introduction

The size and growth rate of nodules are primary indicators used to assess the probability of malignancy and to guide nodule management in accordance with international guidelines [1,2,3,4,5]. These indicate a directly proportional relationship among the initial size, growth rate, and malignancy risk of nodules. The evaluation of nodules, however, encounters several challenges. Due to their high prevalence and typically diminutive size, we commonly use repeat computed tomography (CT) scans to monitor the progression of them. An increase in diagnostic CT scans is associated with a rise in the detection of incidentally identified solitary pulmonary nodules [6,7]. However, we cannot pursue all of these findings because the vast majority of nodules will not indicate lung cancer.

A proper measurement and a clear response from radiology are essential for the clinician to be able to follow the guidelines for incidentally discovered lung nodules. However, numerous studies have demonstrated that radiologists exhibit substantial levels of both intra- and interobserver variability when evaluating lesion sizes, which are attributable to factors such as disagreement in measured CT slices [8,9]. Other sources of variability are related to the scan technique or the patient. For instance, it has been shown that depending on the patient’s breathing pattern, the diameter of the same nodule measured on two successive CT scans (15 min apart) can differ [8]. Variations in the dimensions of the perceived lesion can influence its classification as benign or malignant, thus necessitating a reliable measurement. One idea is that the nodule volume could serve as a more reliable and accurate metric for assessing the lesion, as the approach generates a comprehensive three-dimensional representation of the lesion. In a semi-automatic volumetric method, the radiologist identifies the nodule in question, and the algorithm delineates the lesion’s boundaries and calculates its volume and diameter. The designation “semi-automatic” refers to the radiologist’s ability to modify the lesion delineation if necessary. In past years, semi-automatic measurements have been introduced into a variety of radiology fields [10,11].

Until recently, nodule management has relied on a nodule diameter measurement, despite current guidelines incorporating the nodule volume as a criterion. The British Thoracic Society (BTS) used initial volume and volume doubling time (VDT) estimates alongside the diameter [12], while the Fleischer Society included volume measurements. The volumetric data mainly originate from the Dutch–Belgian Lung Cancer Screening Study (NELSON) evidence [13].

Recent studies indicate that the nodule volume is more stable at lower radiation dose levels than the manual diameter measure, also across different CT scanners and protocols [14,15,16,17]. Han et al. advocated for the utilization of volume instead of diameter to enhance accuracy and reproducibility [14]. Although, a disadvantage that has been pointed out is that the diameter offers a more straightforward interpretation than the volume. Consequently, diameter measurements obtained from the semi-automatic software might also serve as a valuable metric.

Studies that directly compare manual and volumetric measurement methods in the same cohort of nodules, particularly small nodules (below 10 mm), are to date limited. The current study aims to compare the results of semi-automatic diameter and volumetric measurements to those of manual diameter measurements for a series of clinical cases involving only small lung nodules (ranging from around 3 to 10 mm) in order to determine if the semi-automatic measurement methods can lead to an improved consensus between radiologists in diagnostics compared to the current standard measurement method.

## 2. Materials and Methods

### 2.1. Study Group

The study is retrospective in nature and uses paired data from the radiological patient database. The Regional Ethical Review Board approved the study (dnr. 1905454). A total of 414 examinations were obtained by searching the radiological information system (RIS) database for all CT thorax, CT thorax with intravenous contrast, and CT thorax and upper abdomen procedures carried out approximately during the course of a month.

Of these, 237 examinations contained findings other than nodules and tumors (infection, trauma, normal findings in the thorax, chronic lung illness, pleural fluid, and a duplicate ultrasound) and they were therefore not included in the study. A minimum of two CT scans of patients without current cancer treatment were necessary, as a following study will assess changes in lesion growth using the existing semi-automatic approaches. Patients undergoing treatment or with disease control after treatment were therefore excluded, including those with known lung cancer in treatment or follow-up (*n* = 35), patients with other known malignancies with findings in the lungs (*n* = 13), patients with newly discovered lung tumors (>3 cm) in the applicable survey (*n* = 5), and those who passed away prior to the start of the study (*n* = 39).

**Figure 1:**
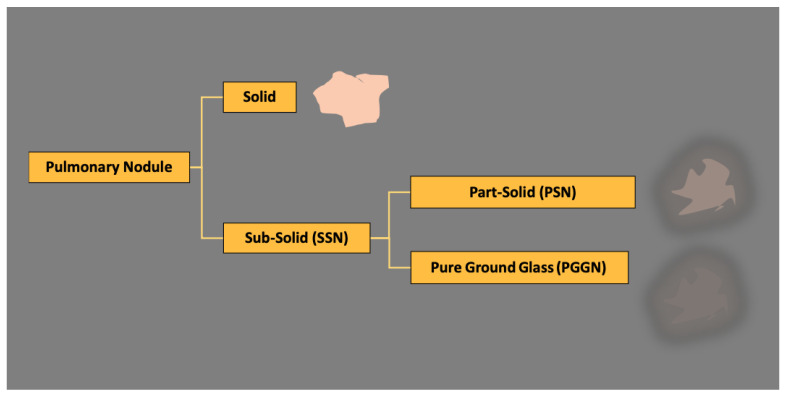
Illustration according to the Fleishner Society of the primary lesion categories, with an emphasis on solid lung nodules measuring roughly 3–10 mm, which are the subject of the current study.

Individuals who were investigated due to pulmonary symptoms (*n* = 29) were excluded in order to have a clinical presentation of accidentally detected lung nodules. Micronodules (less than 3 mm in diameter), nonsolid nodules, part-solid nodules and pleura-based nodules were not included in this interobserver variability study (*n* = 39) because the software is not yet designed to calculate reliable volume estimates for these types of nodules [18,19,20]. Finally, a total of 26 cases of small solid nodules remained for analysis (*n* = 26) (Figure 1). The definite diagnosis of these nodules is presently not known, but is not necessary for the current objective.

### 2.2. Computed Tomography Examination

With the exception of one case, which was carried out on a Siemens CT (Somatom Definition AS+, Siemens Healthcare, Erlangen, Germany), all of the studies were performed using Canon’s CT scanner (Aquilion, Canon Medical Systems, Tokyo, Japan). The standard thoracic CT protocol, which uses dose modulation and modifies the tube current according to patient thickness, was applied, which produces a consistent image quality throughout the 3D volume. Thin CT slices (≤1 mm) were applied, consistent with clinical practice.

### 2.3. Nodule Measurements

#### 2.3.1. The Radiologists Participating in the Study

All radiologists were instructed on how to perform the manual and the semi-automatic measurements. To ensure that all reviewers measured the same lesions, the locations of all nodules were specified beforehand using an electronic mark on the side of the nodule. Nevertheless, the reviewers were free to interpret which plane, in terms of depth, exhibited the largest diameter and proceeded with measurements in that particular slice.

Three radiologists independently measured the nodules in the study material. Two were radiology residents concluding their specialized training and had at least four years of experience in the field of radiology. One of these was specifically focused on thoracic radiology. The third radiologist was a consultant radiologist with extra interest in thoracic radiology who had two years of expertise in the evaluation of thoracic patients at a specialist level. The rationale was to incorporate diverse expertise levels, potentially reflecting a typical general radiology department, to see whether a reduction in variability could be observed. However, it is also known that a greater variability may be expected among less experienced radiologists [21].

First, the manual measurements were performed. The semi-automatic measurements were then performed at least one week later to minimize potential memory effects that may result from a particular measurement. No limit was imposed on the reviewing time.

#### 2.3.2. Manual Standard Measurement

In line with the Fleischner Society’s recommendations for measuring nodules, the manual evaluation was performed using thin slices (<1.5 mm) [22] and an optimal window setting (W/L) for the lungs, along with edge enhancement. The measurements were performed using a SECTRA review PACS and two color-matched 2MP Radiforce Eizo monitors, which had been calibrated prior to the study and are typically used in the clinic. Axial, coronal, and sagittal sections of the chosen nodules, which had been identified beforehand, were accessible. In order to find the average diameter of nodules, the longest diameter (in the axial, coronal, or sagittal plane) was added to the perpendicular short-axis measurement in the same section. Then, these numbers were calculated as in the following example [22]: (4.5 + 3.4)/2 = (5 + 3)/2 = 4 mm.

#### 2.3.3. Semi-Automatic Measurements

**Figure 2:**
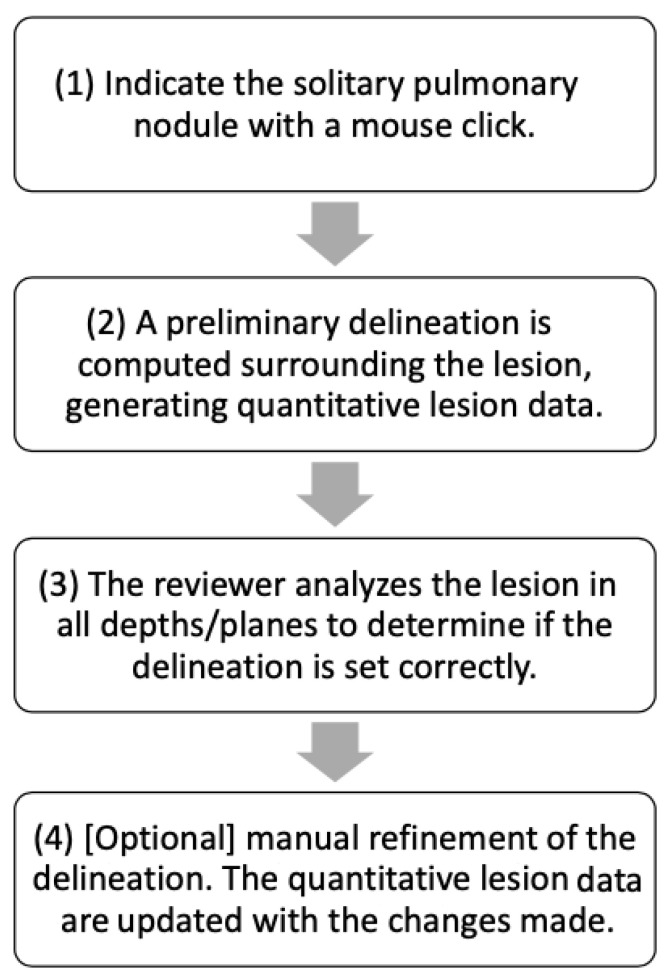
Flowchart illustrating the procedure for semi-automated review.

The volumetric measurement was performed semi-automatically with the Vitrea™ Advanced Visualization application. In the semi-automatic measurement procedure, the computer software delineates the three-dimensional extent of the tumor when the reviewer marks it using the pointer cursor. After that, the radiologist is able to modify the nodule delineation if he or she believes that the software has omitted parts of the nodule or included an excessive amount of the normal tissues surrounding the nodule (Figure 2).

The semi-automatic software (Vitrea™, version 7.15) computes the volume, longest diameter, perpendicular short-axis diameter, and the mean diameter according to the delineation. The maximum short-axis diameter takes the whole volume into account, and the software searches this volume for the longest diameter on the axial plane (according to the Response Evaluation Criteria in Solid Tumors, RECIST) and the perpendicular minimum diameter on that plane [23]. The average diameter utilized in the study is the program-calculated average diameter, where the reviewer is able to make further adjustments (making adjustments in the delineation may also affect the diameter value).

### 2.4. Statistical Analysis

Because the nodule size data have a nonnormal distribution [18,24], Spearman’s test was used to analyze the correlation between the observed values for each method (namely, the conventional manual diameter approach and the semi-automatic approaches, lesion diameter and volume). The test takes into account the sequence in which the reviewers measured the size of the lesion. Spearman’s test coefficient (r) quantifies the statistical dependence between the ranks of two reviewers and is a nonparametric measure of the rank correlation. The range of the correlation coefficient is between 0 and 1, with 0 signifying no link and 1 signifying an ideal association of rankings. Statistical significance is shown when the 95% confidence intervals (95% CIs) do not overlap. The Bland–Altman test was used to visualize the degree of agreement between reviewers for each approach when accounting for relative differences in the measured values [24]. The Bland–Alman method analyzed the dispersion in data points and the limit of agreement for a specific approach (which is a 95% confidence interval) for different measurement sizes, as described more elsewhere [25]. Statistical analyses were carried out using GraphPad (Version 9.00, GraphPad Software, La Jolla, CA, USA). For both statistical tests, the plots were automatically generated by the software.

For each of the three measurement approaches, the number of nodules in which the three reviewers had different measured values was computed. The exact values in submillimeters are used in the statistical tests, as well as in the graphs, for the purpose of measuring variability. In the analysis of discrepant cases, the measurements were rounded to the nearest millimeter, consistent with clinical practice. In addition, the number of nodules that exhibited the highest discrepancy (mm) was calculated. Potential reasons for the discrepancies were analyzed.

## 3. Results

### 3.1. Correlation and Agreement Between Observers

#### 3.1.1. Spearman’s Test

**Figure 3:**
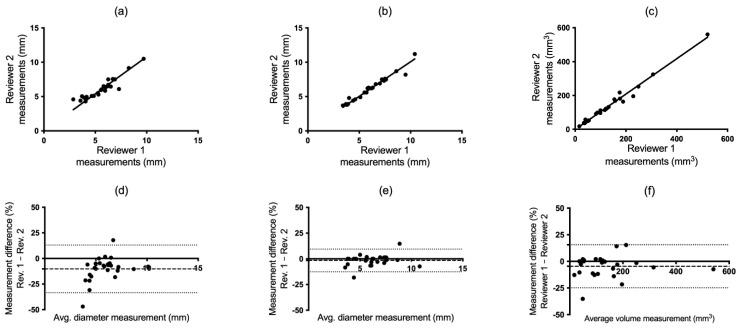
Spearman and Bland–Altman plots demonstrating agreement between reviewers 1 and 2 in manual (**a**,**d**) and semi-automatic (**b**,**e**) mean diameter and volumetric measurements (**c**,**f**).

**Figure 4:**
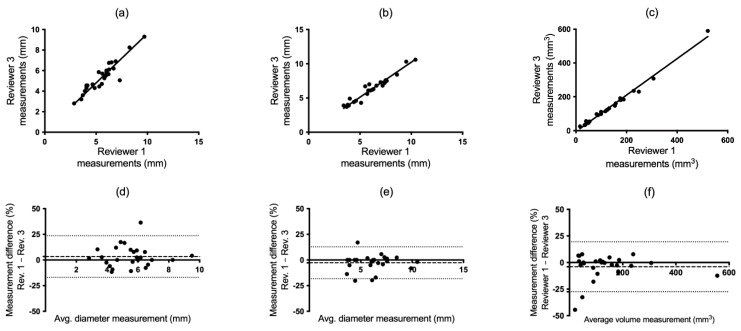
Spearman and Bland–Altman plots demonstrating agreement between reviewers 1 and 3 in manual (**a**,**d**) and semi-automatic (**b**,**e**) mean diameter and volumetric measurements (**c**,**f**).

**Figure 5:**
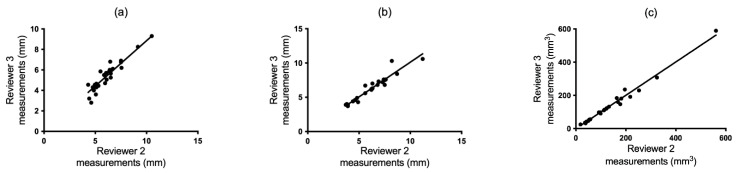

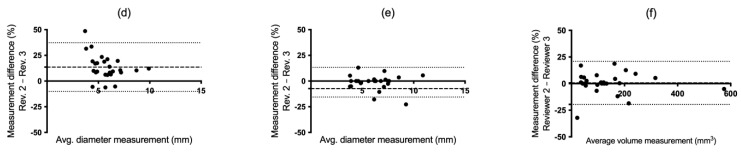
Spearman and Bland–Altman plots demonstrating agreement between reviewers 2 and 3 in manual (**a**,**d**) and semi-automatic (**b**,**e**) mean diameter and volumetric measurements (**c**,**f**).

Spearman’s test provides a measure of whether the reviewers rank the nodules equally in order of size. The Spearman’s test plots for the manual diameter, semi-automatic diameter, and semi-automatic volumetric measurements are presented in Figure 3a–c, Figure 4a–c and Figure 5a–c. Table 1 presents a summary of the interobserver correlation coefficients, which were elevated for all reviewers when measurements were conducted semi-automatically. Statistically significant improvement was observed in two out of three reviewer comparisons for the semi-automatic volumetric measure when compared to manual measures, as indicated by the non-overlapping 95% confidence intervals (Table 1; for R1 vs. R3 and R2 vs. R3). The correlation between semi-automatic diameter measurements was significantly improved in one case (for R1 vs. R2) compared to manual diameter measurements.

#### 3.1.2. Bland–Altman Test

The Bland–Altman (B-A) plots visualize how the differences in the measurements are distributed for different sizes of the measurements and yields confidence intervals (CIs) for the mean error (Figure 3d–f, Figure 4d–f and Figure 5d–f). The B-A plots yielded narrower CIs (and less bias; dashed line) for the semi-automatic diameter method in comparison to the manual measurements for all reviewer comparisons. The semi-automatic volumetric method yielded diverging results in comparison to the manual diameter method. The CIs were comparable in size in one case (Figure 3d,f), narrower in the second case (Figure 4d,f) and larger in the third case (Figure 5d,f). In the manual measurements, reviewer 1 typically interpreted and measured the nodule size as smaller more frequently in comparison to reviewers 2 and 3 (Figure 3d and Figure 5d).

### 3.2. Assessment of Discrepant Cases

**Figure 6:**
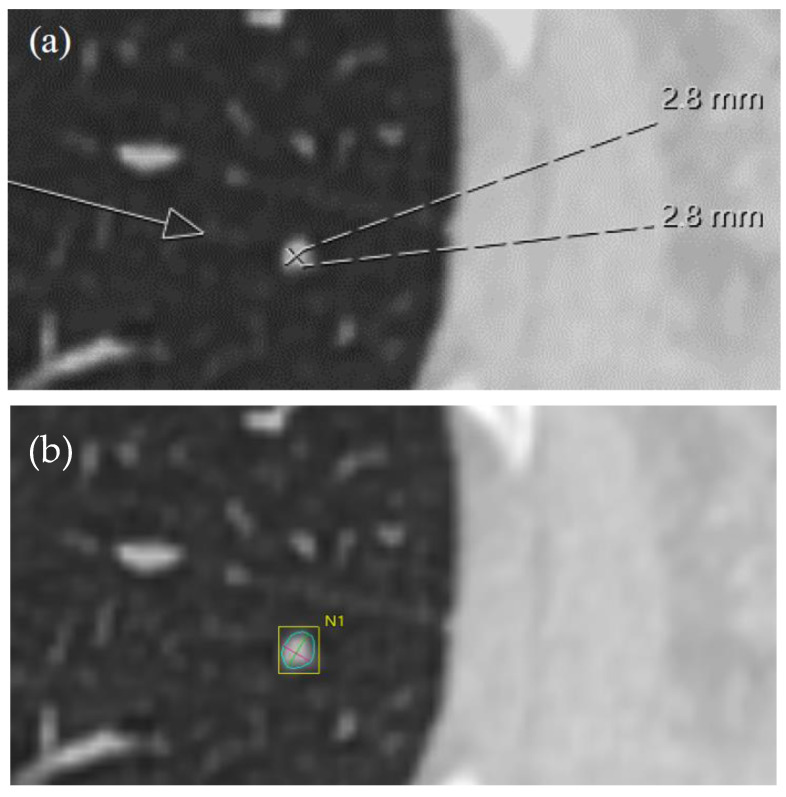
One of the smaller nodules in the study, which is shown by the white arrow, exhibited a notable size discrepancy as assessed by reviewer 3. The manual measurement (**a**) was 2.8 × 2.8 mm (white lines), while the semi-automatic measurement (**b**) yielded greater values of 3.7 × 3.2 mm (the blue line denotes the longest diameter; while the red line denotes the smallest diameter).

The measured sizes of the 26 nodules varied over the spectrum of the selection (ranging from 3 mm to 11 mm) when accounting for both the manual and the semi-automatic diameter methods. The number of nodules in which the three reviewers had different measured values was substantially greater when the diameter was measured manually (17 of 26; 68%) compared to the semi-automatic diameter measurement (9 of 26; 36%). The number of nodules that exhibited the highest discrepancy (2 mm) among the radiologists was five when using manual measurement and only one when employing the semi-automatic diameter measurement. Two of these five nodules were very small, the smallest in the study material, as seen in Figure 6.

Two of the other nodules with a relatively high discrepancy were highly irregular, where one nodule was also the most difficult to measure (spiculated, irregular) using the semi-automated diameter measurement technique (this was the only case that exhibited a 2 mm inter-reviewer discrepancy) (Figure 3). The sixth nodule was one of the largest in the study material, with a slightly irregular shape. All three measures were performed in the same plane in 12 of the 25 nodules. In over half of the measurements, the reviewers could not agree on which plane had the largest diameter.

**Figure 7:**
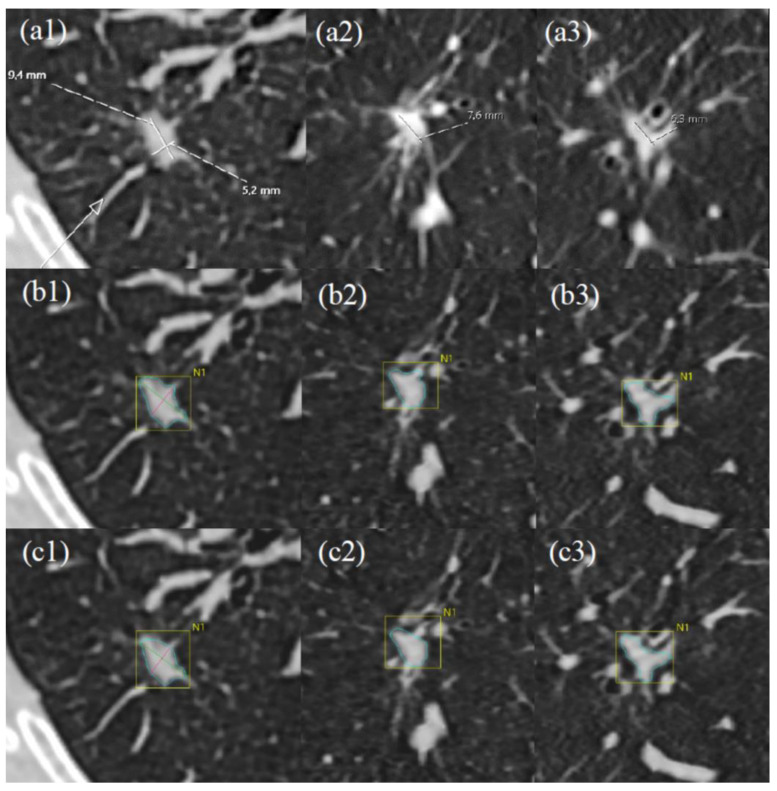
A prominently discernible spiculated nodule. Reviewer 1 performed manual measurements in axial (**a1**), coronal (**a2**), and sagittal (**a3**) sections. The largest diameter was measured in the axial section (**a1**) and was found to be 9.4 × 5.2 mm. Reviewer 1 utilized the semi-automatic software (Vitrea™) (**b1**–**b3**) to measure the same nodule and obtained a volume of 236.4 mm^3^ (diameter: 10.3 × 7.7 mm) (**b1**–**b3**). Subsequently, following modifications in the nodule delineation (**c1**–**c3**), the volume measured 228.6 mm³ (diameter: 9.5 × 6.8 mm).

**Figure 8:**
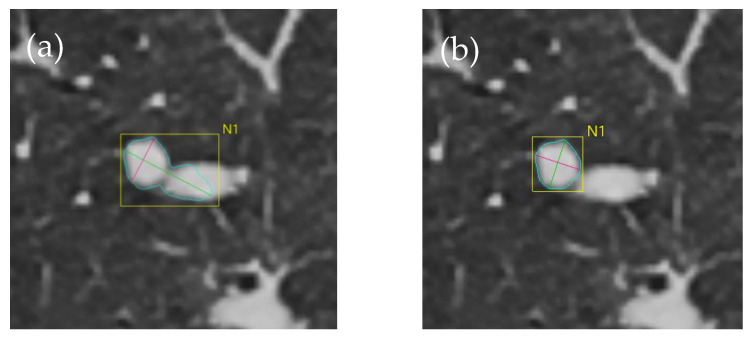
A complicated situation where the nodule partially blended together with blood vessels in certain CT slices. This caused the delineation to be elongated and partially incorrect, where both the nodule and parts of the blood vessel (on the right) were included in the initial estimate made by the semi-automatic algorithm ((**a**); volume: 431 mm^3^, dimensions: 11.2 × 7.5 mm; blue-colored lines represent the longest diameter). The reviewers adjusted the delineation to accurately depict the size of the nodule ((**b**); reviewer 3 after adjustments; volume: 142.8 mm^3^, diameter: 7.6 × 7.3 mm).

Overall, according to the resulting delineation, 18 of the nodules in the whole study material had relatively rounded boundaries in the edge characteristics, typically resembling the nodule depicted in Figure 6. Four of the nodules exhibited modest irregularity, while three nodules were highly spiculated (Figure 7). Figure 8 shows an example of a case with a substantial difference between the initial estimate by the semi-automatic algorithm and the final estimate by a reviewer.

#### Volume

In the semi-automatic volume measurements, the nodule volumes ranged from a minimum of 16 mm^3^ to a maximum of 590 mm^3^. There were three nodules where all three reviewers arrived at the same measurements and seven nodules that differed by five mm^3^. A further five nodules differed by more than 20 mm^3^, and they all had some degree of irregularity, ranging from barely irregular to plainly spiculated (Figure 4). The reviewers had the possibility to modify the outlines of the nodules. The number of times modifications were made in nodule delineation varied significantly among reviewers. The three reviewers opted not to make any further adjustments of two (reviewer 1), seven (reviewer 2), and sixteen (reviewer 3) of the twenty-six measured nodules.

## 4. Discussion

The present study focused on small nodules (∼3–10 mm) and revealed that semi-automatic processes exhibited lower interobserver variability compared to the manual measuring method, with remarkably high (almost perfect) correlation coefficients observed for both the mean diameter and volume. This was particularly apparent in Spearman’s correlation test, which took into consideration the arrangement of the lesions based on the perceived size. The volumetric technique was determined to be markedly superior to the manual method for every reviewer comparison (Table 1). Various reviewers assessed each strategy, and the comparisons yielded consistent results for each approach (e.g., comparable correlation coefficients), with minimal variation (Table 1).

According to Spearman’s correlation test, the semi-automatic volumetric approach performed comparably or in some cases slightly better than the semi-automatic diameter method (Table 1). However, it should be noted that these two measures are not independent of each other. Alterations in the delineation of the volume including the nodule can potentially impact the diameter measure. The Bland–Altman (B-A) test accounts for the relative differences between the measured values. The B-A graphs also demonstrated a greater agreement among the reviewers when employing the semi-automatic diameter measurement as opposed to the manual diameter measurement (Figure 3, Figure 4 and Figure 5). This is reasonable, since the manual technique depends on several distinct procedures and judgments before the actual measurement, where each step has the potential to introduce variability.

On the other hand, the semi-automatic measurement method showed more scattered results for volume in the B-A analysis. Due to the increased sensitivity of the volumetric approach, it is anticipated that not all values may be measured with good precision. The surprising finding was that several nodules exhibited a difference of over 25% (Figure 3f, Figure 4f and Figure 5f). A possible reason for these results is that a three-dimensional measurement that takes into account variations in the entire three-dimensional shape of the nodule (x, y, z) may show greater variations compared to a measurement that encompasses only two planes. In addition, when evaluating small nodules, differences that are small in absolute terms can be large in relation to the size of the nodule itself and thus have a large impact on the B-A test.

Despite the prior identification and indication of all nodules in the study, the reviewer must ascertain the precise plane and location that exhibits the greatest diameter. Reviewers measured the “longest” diameter on different planes in around half of the study cases. Precise delineation of the nodule borders is crucial in the volumetric approach, and no adjustments are necessary if the radiologist concurs with the software’s delineation. However, reviewer 3 did not modify the delineation in 16 of the 26 nodules, whereas the other two reviewers made adjustments in the majority of cases. This finding could be a significant secondary finding. One could consider the reviewers’ individual characteristics as a factor, specifically the limit or threshold that each individual deems to be “good enough”. This is a subjective factor, likely related to how thorough a reviewer is or how thorough a reviewer strives to be. Prior to the trial, each reviewer was instructed to make any necessary adjustments to the delineation in order to precisely surround the nodule. The influence of these instructions on the study outcomes is uncertain, but increased understanding and knowledge among reviewers about the importance of this step should help to reduce observer variability even further. When novel procedures are implemented, it is well understood that learning curves can take weeks to years to level off, meaning that the semi-automated tool may achieve even lower interobserver variability.

Across all methods, the largest interobserver discrepancies were found for some of the smallest nodules and the very irregular-shaped nodules. This is in good agreement with what previous studies have shown: small and/or irregular nodules show greater interobserver variability than larger and/or more evenly demarcated nodules, both with manual measurement methods and with volumetric methods [18,19,22,26]. At the same time, several of the smaller nodules in the study showed very good agreement between the reviewers, and so it can be assumed that several factors in combination may be at play for each individual nodule.

A limitation of the study is that the study material was relatively small (*n* = 26), but the design was paired (i.e., the reviewers measured the same cases for each method), which increases the statistical power. The differences that were found in the comparisons also show that the number of cases was sufficient to demonstrate that there was an effect.

The manual method is currently the gold standard, regardless of previous research on manual and semi-automatic measurement techniques. Despite the positive outcomes discovered in the present study, semi-automatic measurement techniques nevertheless possess certain drawbacks. The main problem stems from the operational strategy, which entails detecting attenuation differences between the nodule and the surrounding tissues. When dealing with minor attenuation differences, for example, in subsolid nodules and nodules near arteries and pleura, the software may struggle to accurately outline these differences [19]. Therefore, these situations rely heavily on the adjustments made by the reviewer. Moreover, it is relevant to acknowledge that variances may exist among different software. A study comparing programs developed by different manufacturers found volume differences of up to 50% for nodules measuring 100 mm^3^. As a consequence, it is crucial to monitor nodules using the identical software. In the near future, software will likely be improved by implementing artificial intelligence (AI)-based algorithms that can better deal with difficult scenarios, where the nodule partially blends into the surrounding tissues or where there are simply limited attenuation differences in the lesions’ edge characteristics. As such, AI-based methods may lead to an even more accurate delineation of challenging cases before adjustments are made by the reviewer.

In conclusion, a low inter- and intra-observer variability assessment method will provide safer treatment recommendations, lowering the risk of unnecessary invasive measures or, conversely, missing an early-stage malignancy. Reducing measurement variability would lead to more equal patient treatment and increased cost-efficiency. There is mounting evidence that semi-automatic volumetric measurements are more precise and repeatable than manual diameter measurements [8]. It has also been demonstrated that volumetric approaches have superior measurement reliability in terms of size progression or regression compared to manual diameter measurement [8]. Previous studies have, however, almost exclusively compared measurements of larger nodules. The present study shows that using semi-automatic measures solely for small nodules (∼3–10 mm) results in improved correlations and reduced variability among radiologists compared to the existing standard measurement method. As lung cancer screening programs are adopted, semi-automatic measuring should be able to aid in the more precise differentiation of malignant and benign nodules and to reduce the variability between reviewers, as shown in the present study.

## Figures and Tables

**Table 1:** A summary of the Spearman’s correlation test * results for different reviewer comparisons ^¶^ when applying the three different measurement methods.

	Manual Measurement	Semi-Automatic Measurement	
Comparison	Avg. Diameter [95% CI]	Avg. Diameter [95% CI]	Volume [95% CI]
R1 vs. R2	0.933 [0.851; 0.966]	0.986 [0.967; 0.994]	0.980 [0.955; 0.991]
R1 vs. R3	0.903 [0.788; 0.957]	0.954 [0.897; 0.980]	0.989 [0.975; 0.995]
R2 vs. R3	0.906 [0.794; 0.956]	0.962 [0.913; 0.983]	0.988 [0.973; 0.995]
Avg. [Stdev.]	0.914 ± 0.017	0.967 ± 0.017	0.986 ± 0.005

* Spearman’s correlation coefficient has a range of 0 to 1, with 0 indicating no relationship and 1 indicating a perfect association of ranks. ^¶^ Different reviewer comparisons, for instance, R1 vs. R3, correspond to the comparison between reviewer 1 and reviewer 3.

## Data Availability

Data are available by contacting the corresponding author (TS).
